# Long Neurocognitive and Neuropsychiatric Sequelae in Participants with Post-COVID-19 Infection: A Longitudinal Study

**DOI:** 10.3390/neurolint16040064

**Published:** 2024-08-16

**Authors:** Marta Almeria, Juan Carlos Cejudo, Joan Deus, Jerzy Krupinski

**Affiliations:** 1 Department of Neurology, MútuaTerrassa University Hospital, 08221 Barcelona, Spain; jkrupinski@mutuaterrassa.es; 2 Cognitive Impairment and Dementia Unit, Hospital Sagrat Cor—Hermanas Hospitalarias, 08760 Barcelona, Spain; jccbnps@yahoo.es; 3 Clinical and Health Department, Psychology Faculty, Autonomous University of Barcelona, 08193 Barcelona, Spain; 4 MRI Research Unit, Department of Radiology Hospital del Mar, 08003 Barcelona, Spain; 5 Life Sciences Department, Manchester Metropolitan University, Manchester M15 6BH, UK

**Keywords:** long COVID-19, cognition, subjective cognitive complaints, persistent symptoms

## Abstract

Objective: To evaluate and characterize the cognitive changes in COVID-19 participants at 6-month follow-up, and to explore a possible association with clinical symptoms, emotional disturbance and disease severity. Methods: This single-center longitudinal cohort study included participants aged 20 and 60 years old to exclude cognitive impairment age-associated with confirmed COVID-19 infection. The initial evaluation occurred 10 to 30 days after hospital or ambulatory discharge, with a subsequent follow-up at 6 months. Patients who had a history of cognitive impairment, neurological conditions, or serious psychiatric disorders were not included. Information on demographics and laboratory results was gathered from medical records. Cognitive outcomes were assessed with a neuropsychological battery including attention, verbal and visual memory, language and executive function tests. Results: A total of 200 participants were included in the study, and 108 completed the follow-up visit. At the 6-month follow-up, comparing the means from baseline with those of the follow-up evaluation, significant overall improvement was observed in verbal and visual memory subtests (*p* = 0.001), processing speed (*p* = 0.001), executive function (*p* = 0.028; *p* = 0.016) and naming (*p* = 0.001), independently of disease severity and cognitive complaints. Anxiety and depression were significantly higher in groups with Subjective Cognitive Complaints (SCC) compared to those without (*p* < 0.01 for both). Conclusions: Persistent symptoms are common regardless of disease severity and are often linked to cognitive complaints. Six months after COVID-19, the most frequently reported symptoms included headache, dyspnea, fatigue, cognitive complaints, anxiety, and depression. No cognitive impairment was found to be associated with the severity of COVID-19. Overall, neuropsychological and psychopathological improvement was observed at 6 months regardless of disease severity and cognitive complaints.

## 1. Introduction

SARS-CoV-2, the virus responsible for coronavirus disease 2019 (COVID-19), can lead to a wide range of neurological complications [1,2,3]. Among these, cognitive complaints following clinical recovery from acute respiratory symptoms are particularly common [4]. Approximately 50% of infected patients experience symptoms later in the post-acute phase [5,6,7], and around 45% of survivors report persistent symptoms four months after infection. Fatigue is the most frequent both in hospitalized and non-hospitalized patients [6]. Morin et al. [8] found that 51% of 244 post-hospitalization COVID-19 patients reported at least one new symptom at 4 months that had not been present in the acute phase of infection, including fatigue (31%), neuropsychological symptoms (21%), and dyspnea (16%). Up to 62 symptoms were identified as associated with infection with COVID-19 infection after 12 weeks [9].

Overall, fatigue, dyspnea, sleep disturbances, and neuropsychological impairment are the most reported symptoms [10,11,12]. Indeed, neurocognitive impairment and psychological symptoms, including “brain fog,” became especially prevalent after 6–12 months [13]. Almeria et al. [14,15,16] found that cognitive complaints are frequent and mostly associated with higher rates of anxiety and depression. Similarly, Poletti et al. [17] found that the factor that most affected cognitive performance was depressive psychopathology. Up to one in three patients experience neuropsychological deficits for weeks or even months [18], with neurocognitive alterations being more frequent than reported in other viral processes. Neurocognitive impairment is frequently highlighted in long-COVID research [19,20,21,22]. In this scenario, COVID-19 could have harmful consequences even after the post-acute phase, depicting a new pathological condition: the “post-COVID-19 syndrome (PCS)” or “long COVID” [23]; that is, as a complex of signs and symptoms that could not be explained by other diagnoses that last more than 12 weeks after COVID-19.

Michelsen et al. [24] conducted a systematic review with 39 studies, revealing considerable diversity in the potential risk factors for developing long COVID. The authors attributed this multiplicity to variations in study design, sample size, and follow-up time, among others, complicating a thorough understanding of the syndrome [21]. Therefore, it is important to assess whether different clinical aspects of COVID-19 are linked to a higher risk of long-term cognitive impairment, as this could enable clinicians to predict which patients are at increased risk of developing such complications.

To our knowledge, few studies with small samples have assessed the progression of cognitive impairment six months after hospital discharge following a comprehensive baseline neuropsychological assessment. Understanding the progression of cognitive alterations is critical, as it serves an important indicator of functional recovery. Indeed, while most COVID-19 patients recover within months of hospitalization, persisting cognitive and psychopathological symptoms can impact negatively on their quality of life. Our aim was to characterize the cognitive and clinical changes of COVID-19 patients at a six-month follow-up and investigate their potential relationship with clinical symptoms, emotional issues, and the severity of the disease.

## 2. Methods

### 2.1. Study Design and Participants

This is a longitudinal, consecutive case study involving adult patients evaluated at Hospital Universitari MútuaTerrassa (HUMT) from April 2020 to February 2022. All patients had SARS-CoV-2 infection confirmed by positive polymerase chain reaction (PCR) from nasopharyngeal swab or by positive serology. Participants were aged between 20 and 60 years, with those over 60 years excluded to avoid age-related cognitive decline. Patients with prior cognitive impairment and any other nervous system manifestation or severe psychiatric disorders with potential cognitive deficits were excluded. Assessment was conducted between 10 and 30 days after hospital or ambulatory discharge following COVID-19 infection, with follow-up at 6 months (±15 days). The study was approved by the local ethic committee, and all subjects provided informed consent.

### 2.2. Data Collection and Definitions

Data were obtained from the HUMT database by conducting a retrospective analysis of electronic health records. We collected and analyzed information on demographic details, existing comorbidities, blood test results (such as ferritin and D-Dimer), as well as symptoms and signs at the time of presentation and after six months. To evaluate cognitive complaints, participants were asked whether they had experienced any changes in their cognitive function though an interview. To assess cognitive impairment, a specific neuropsychological battery was administered for this population. These assessments were carried out by the same neuropsychology expert in a one-hour session. All tests were validated for our population and are recognized for international use. The battery included the Test de Aprendizaje Verbal España Complutense Complutense (TAVEC) [25], Visual Reproduction of the Wechsler Memory Scale IV (WMS-IV) [26], Digits forward and Backward, Letter and Numbers, Trail Making Test (TMT A and B), SDMT, Stroop, phonemic and semantic fluency, and Boston Naming Test from the NEURONORMA project (NN) [27,28,29,30,31,32]. Scores were standardized according to local normative data, accounting for age and education, using the T-score (PT) (mean 50 points and SD of 10 points). The Hospital Anxiety and Depression Scale (HAD) [33] was administered to assess anxiety and depression symptoms.

### 2.3. Statistical Analysis

The sample was classified into four groups according to the severity of illness, which was determined by the need for hospitalization, oxygen therapy, and ICU admission: no hospitalization (NH, *n* = 21); hospitalized patients not requiring ICU or oxygen therapy (HOSP, *n* = 42); hospitalized patients needing oxygen therapy but not ICU care (OXY, *n* = 107); and those admitted to ICU (ICU, *n* = 31). In the primary analysis, the descriptive data of the sample were obtained according to the severity groups, clinical symptoms and cognitive complaints. Normality assumptions were checked for all study variables with the Kolmogorov–Smirnov test, assuming a level of significance > 0.05 for the assumption of normality distributions. Descriptive data were obtained for each group attending Subjective Cognitive Complaints (SCC), as well as symptoms at both initial assessment and 6-month follow-up.

In the subsequent analysis, inferential tests were conducted to compare the study variables across the groups. Mean comparison tests were employed for both independent data (between groups) and paired data (baseline versus 6 months). Student’s *t*-test was used for comparisons between two groups when the data followed a normal distribution and showed homogeneity of variance. ANOVA was utilized for comparisons involving more than two groups under similar conditions. Levene’s test was applied to assess the homogeneity of variance for both Student’s *t*-tests and ANOVA. Post hoc ANOVA contrasts were conducted using the Scheffé test. For variables that did not adhere to a normal distribution or had fewer than 30 subjects, the Kruskal–Wallis rank test and Mann–Whitney U test were employed to compare means. The Chi-square test was used for comparing proportions between groups. Statistical analyses were performed using R. CRAN. Oficina de software libre (CIXUG), Spanish National Research Network, http://cran.es.r-project.org/ (accessed on 12 October 2020).

## 3. Results

### 3.1. Demographic and Clinical Characteristics

**Figure 1:**
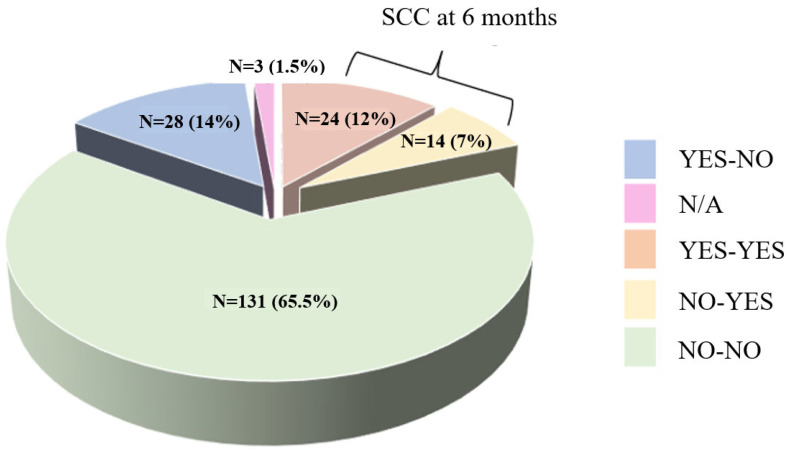
Evolution of subjective cognitive complaints at 6 months.

A total of 200 patients who tested positive for SARS-CoV-2 were initially enrolled in the study. Of these, 108 participants accepted an outpatient reassessment and completed the follow-up at 6 months. Among the 92 participants who discontinued the study, 88 declined further participation and reported no cognitive deficits, 2 were lost to follow-up, 1 passed away, and 1 was diagnosed with human immunodeficiency virus (HIV) during the follow-up. There were 64 males (59.25%) and 44 females (40.75%) in the cohort, with mean age of 49.10 years (SD: 7.67). Among them, 38 (35.18%) reported SCC, while 70 (64.81%) did not. Figure 1 illustrates the evolution of cognitive complaints among the initial sample of 200 subjects.

We aimed to determine whether the persistent symptoms were newly developed or were already part of the disease in the acute phase. Among the 42 participants experiencing fatigue at 6 months, only 1 individual had not reported it during the acute phase of the infection. Similarly, among the 17 with headache, only 1 was newly reported. This pattern was also observed in 1 of the 8 patients with myalgias, 1 out of 49 with anxiety, and 2 of the 17 with dyspnea. In contrast, all patients with persistent cough (*n* = 2), dysgeusia (*n* = 3), anosmia (*n* = 8) and depression (*n* = 38) had experienced these symptoms since the onset of the disease.

### 3.2. Neuropsychological Findings

Comparing the initial assessment with the 6-month follow-up for the entire sample, significant overall improvement was observed in subtests of verbal (*p* = 0.001) and visual (*p* = 0.001) declarative memory, processing speed (*p* = 0.001), executive function (*p* = 0.028; *p* = 0.016) and naming (*p* = 0.001). The effect sizes ranged from small to moderate [range: 0.15–0.68], with the greatest improvement observed in the first trial of verbal memory. All mean scores fell within the normal range. Table 1 displays the scores in the different neuropsychological subtests for the entire sample (*n* = 108). Scores are expressed as direct score.

#### 3.2.1. Neuropsychological Results Depending on the Severity of the Disease

Comparison of baseline versus longitudinal performance across disease severity groups: NH (*n* = 10), HOSP (*n* = 21), OXY (*n* = 56), and ICU (*n* = 21). Table 2 shows the results expressed in direct score of the comparison between the baseline performance and at 6 months for the patients without initial admission. These patients showed a global improvement at 6 months, with statistically significant differences in the first learning trial of memory (*p* = 0.026), immediate recall with semantic clues (*p* = 0.043) and a significant reduction in anxiety (*p* = 0.007), with a high size effect [range: 0.69–0.90].

Table 3 presents the results in raw scores comparing baseline and 6-month performance for hospitalized patients without oxygen therapy. Significant improvements were noted at 6 months, particularly in initial learning trial (*p* = 0.003), total recall (*p* = 0.002), immediate recall (*p* = 0.033), and semantic cue recall (*p* = 0.009), with large effect sizes ranging from 0.46 to 0.93.

Table 4 shows the results expressed in direct score of the comparison between baseline performance and at 6 months for patients hospitalized with oxygen. In this group, a global improvement was observed at 6 months, with statistically significant differences in verbal declarative memory (*p* = 0.001, *p* = 0.0002, *p* = 0.003, *p* = 0.008) and visual (*p* = 0.001, *p* = 0.003), working memory (*p* = 0.038), processing speed (*p* = 0.001) and language (*p* = 0.035, *p* = 0.022), with a size effect between small and moderate [range: 0.18–0.63].

Table 5 shows the results expressed in direct score of the comparison between baseline performance and at 6 months for patients who required admission to the ICU. In this group, a global improvement was observed at 6 months, with statistically significant differences in verbal (*p* = 0.002) and visual declarative memory (*p* = 0.032), working memory (*p* = 0.008), cognitive flexibility (*p* = 0.027) and naming (*p* = 0.001), with a moderate size effect [range: 0.29–0.52].

#### 3.2.2. Neuropsychological Results Based on Cognitive Complaints

Baseline versus longitudinal performance was compared based on cognitive complaints. Initially, we observed the group of patients who did not present SCC at 6 months (*n* = 70). Table 6 illustrates the comparison between baseline performance and follow-up for the subgroup without SCC. A global improvement was observed, mainly in verbal (*p* = 0.001, *p* = 0.002) and visual (*p* = 0.001, *p* = 0.006) declarative memory and naming (*p* = 0.005), with a small to moderate effect size [range: 0.14–0.551].

Table 7 shows the longitudinal follow-up for subjects with SCC (*n* = 38), revealing a significant improvement in verbal (*p* = 0.001) and visual (*p* = 0.001) declarative memory, processing speed (*p* = 0.001), executive function (*p* = 0.011, *p* = 0.036) and naming (*p* = 0.001), with a moderate to high effect size [range: 0.27–0.94].

When comparing the baseline assessments of subjects without SCC (*n* = 70) to those with SCC (*n* = 38), there were no significant differences (*p* > 0.05) except for anxiety (t: −6.01; *p* = 0.001) and depression (t: −6.39; *p* = 0.001), which were significantly higher in the group with cognitive complaints.

For subjects with SCC (*n* = 70), four groups were established based on the conversion between the baseline examination and the follow-up at 6 months, as follows: patients without initial complaints and without complaints at 6 months: No–No, *n* = 54 (Group 0); patients with initial complaints and no complaints at 6 months: Yes–No, *n* = 16 (Group 1); patients without initial complaints and with complaints at 6 months: No–Yes, *n* = 16 (Group 2); and patients with initial complaints and at 6 months: Yes–Yes, *n* = 22 (Group 3). Tables S1–S4 present the neuropsychological results at baseline and at 6 months for each of the group.

For patients without any cognitive complaints at baseline or follow-up (Table S1), a global improvement was observed, mainly in verbal (*p* = 0.001, *p* = 0.002, *p* = 0.039) and visual (*p* = 0.001) declarative memory, processing speed (*p* = 0.004, *p* = 0.029), executive function (*p* = 0.002) and language (*p* = 0.026, *p* = 0.002), with effect sizes ranging from low and moderate–high [range: 0.13–1].

For patients who had cognitive complaints initially but not at follow-up (Table S2), improvements were noted in verbal declarative memory (*p* = 0.006, *p* = 0.010, *p* = 0.022, *p* = 0.018, *p* = 0.001, *p* = 0.008), processing speed (*p* = 0.015) and naming (*p* = 0.033). Additionally, there was a decrease in anxiety (*p* = 0.004) and depression (*p* = 0.016), with a moderate–high to very high effect size for anxiety and depression [range: 0.27–1].

For patients who did not have previous complaints but developed them subsequently (Table S3), there were no statistically significant differences except for an increase in anxious symptoms (*p* = 0.007), with a moderate effect size (d = 0.49).

For patients who had both previous and subsequent complaints (Table S4), there was a noticeable overall improvement, particularly in verbal declarative memory (*p* = 0.001, *p* = 0.007, *p* = 0.006, *p* = 0.049, *p* = 0.008) and visual memory (*p* = 0.047, *p* = 0.001), as well as in processing speed (*p* = 0.008) and language (*p* = 0.049, *p* = 0.009). The effect sizes ranged from low to moderate–high, with a particularly significant effect observed in the first trial of verbal memory (range: 0.26–0.81). However, anxiety and depression scores remained high, exceeding the cutoff point, showing no significant change.

Comparison of the 6-month evaluations among subgroups (0, 1, 2, and 3) revealed that Group 2 (No–Yes) exhibited significantly lower performance in verbal declarative memory subtests (*p* = 0.001, *p* = 0.004, *p* = 0.002, *p* = 0.009), executive function (*p* = 0.003), processing speed (*p* = 0.002, *p* = 0.001), working memory (*p* = 0.003), language (*p* = 0.003), anxiety (*p* = 0.001), and depression (*p* = 0.017) compared to Group 0 (No-No). Table S5 illustrates the subtests where performance differed between the groups. No significant differences were observed between the groups without cognitive complaints at 6 months (Groups 0 and 1). Significant differences were noted in terms of anxiety and depression between groups with and without cognitive complaints (*p* > 0.05), with a very high F value in ANOVA [13.23 and 16.72], showing significantly higher levels in the groups with SCC (*p* < 0.01 and *p* < 0.01), respectively.

#### 3.2.3. Neuropsychological Results Based on Clinical Symptoms

Considering the relationship between persistent symptoms (fatigue, anxiety and depression) and cognitive complaints, we evaluated the presence of persistent symptoms. It was evaluated based on the evolution of cognitive complaints across established subgroups based on cognitive complaints. Table S6 shows an association between the groups presenting cognitive complaints at 6 months and the persistence of symptoms. On the contrary, the groups without complaints had a lower proportion of patients with persistent symptoms (Chi^2^: 24.17, *p* = 0.001).

Patients with persistent fatigue showed a general improvement in neurocognitive performance, with significant differences in TAVEC-1, with a mean of 6.14 (SD: 1.88) vs. 7.57 (SD: 1.81) (t = −4.18, *p* = 0.001); in TAVEC-Total, with a mean of 51.19 (8.88) vs. 56.10 (SD: 10.32) (t = −4.15, *p* = 0.001); TAVEC-IMR, with a mean of 10.69 (SD: 2.78) vs. 11.86 (SD: 2.96) (t = −3.59, *p* = 0.001); TAVEC-IMRSC, with a mean of 11.71 (SD: 2.60) vs. 13.14 (SD: 2.57) (t = −4.12, *p* = 0.001); WMS-IMR, with a mean of 32.93 (SD: 6.49) vs. 34.95 (SD: 5.50) (t = −3.53, *p* = 0.001); WMS-DFR, with a mean of 24.78 (SD: 9.66) vs. 28.78 (SD: 8.58) (t = −3.07, *p* = 0.004); WMS-REC, with a mean of 5.00 (SD: 1.72) vs. 5.57 (SD: 1.62) (t = −2.44, *p* = 0.019); reverse digits, with a mean of 4.10 (SD: 1.24) vs. 4.43 (SD: 1.15) (t = −2.32, *p* = 0.025); SDMT, with a mean of 39.02 (SD: 11.48) vs. 41.79 (SD: 12.43) (t = −2.94, *p* = 0.005); and BNT, with a mean of 48.76 (SD: 6.76) vs. 50.07 (SD: 6.20) (t = −2.87, *p* = 0.006).

Patients with persistent anxiety obtained a general improvement in neurocognitive performance, with significant differences in TAVEC-1, with a mean of 6.53 (SD: 1.97) vs. 7.41 (SD: 1.81) (t = −3.27, *p* = 0.002); in TAVEC-IMR, with a mean of 10.96 (SD: 2.69) vs. 11.80 (SD: 3.07) (t = −2.73, *p* = 0.009); TAVEC-IMRSC, with a mean of 12.00 (SD: 2.55) vs. 12.96 (SD: 2.74) (t = −2.93, *p* = 0.005); WMS-DFR, with a mean of 26.16 (SD: 28.82) vs. 28.82 (SD: 8.85) (t = −2.44, *p* = 0.018); BNT, with a mean of 49.33 (SD: 7.43) vs. 50.29 (SD: 7.15) (t = −2.21, *p* = 0.032); and a worsening in HAD-A, with a mean of 9.88 (SD: 3.78) vs. 11.39 (SD: 3.14) (t = −2.84, *p* = 0.006).

Patients with persistent depression obtained a general improvement in cognitive performance, with significant differences in TAVEC-1, with a mean of 6.39 (SD: 1.89) vs. 7.08 (SD: 1.74) (t = −2.09, *p* = 0.043); WMS-DFR, with a mean of 23.97 (SD: 9.06) vs. 27.42 (SD: 8.84) (t = −2.60, *p* = 0.013); a worsening in HAD-A, with a mean of 10.05 (SD: 3.90) vs. 11.42 (SD: 3.67) (t = −2.16, *p* = 0.037); and a worsening inHAD-D, with a mean of 8.21 (SD: 3.70) vs. 10.05 (SD: 2.73) (t = −2.81, *p* = 0.008).

## 4. Discussion

Our study was designed to evaluate and characterize the cognitive changes of COVID-19 participants at 6-month follow-up.

### 4.1. Neuropsychological Outcomes

#### 4.1.1. Illness Severity

We found no correlation between disease severity and impaired neurocognitive performance. All groups, irrespective of the severity of the disease, exhibited scores within the normal range and demonstrated improvements from the baseline assessment. These results are consistent with other studies that similarly reported no relationship between disease severity and neuropsychological performance [15,16,33,34]. Although no differences were observed in neurocognitive performance, non-hospitalized patients exhibited higher levels of anxiety and depression in the initial phase of the disease. These patients showed significant improvement in anxiety at 6 months, though their levels remained higher than those of the other groups. It is expected that patients with more severe illness will exhibit increased anxiety and depressive symptoms. However, this symptomatology had previously been reported in non-hospitalized patients [14,15,16], possibly due to the awareness of their illness’s severity and potential worsening, especially during the pandemic’s early phase when hospital care was limited.

#### 4.1.2. Subjective Cognitive Complaints

Given that the onset of cognitive complaints occurred at different phases of the disease, we evaluated the differences based on the evolution of the SCC by establishment of four groups (No–No; Yes–No; No–Yes; Yes–Yes). A global improvement was observed in all groups at the neuropsychological follow-up, primarily in memory, speed processing, executive function, and language tasks. However, the group that initially had no complaints but developed them at 6 months (No–Yes conversion) did not show this improvement at follow-up and exhibited lower performance compared to the group with no cognitive complaints. Interestingly, this group had higher scores in anxiety and depression, suggesting that this symptomatology could influence the lack of neurocognitive improvement at 6 months. The association between SCC and an increase in psychopathology had already been previously described [14,15,16].

On the contrary, patients who initially presented cognitive complaints but did not have them at follow-up (Yes–No conversion) showed cognitive and psycho-affective improvements. Our results point that anxiety and depression are the indicators that differentiate the groups with cognitive complaints (No–Yes, Yes–Yes) from those without (No–No, Yes–No), strengthening the association between cognitive complaints and psychopathological symptoms, but not with neurocognition performance [20,35].

#### 4.1.3. Persistent Clinical Symptoms on Follow-Up

In our sample, the occurrence of persistent symptoms at 6 months was frequent, consistent with the existing literature that shows a significant proportion of individuals experience symptoms lasting from several weeks to months [5,6,7]. A substantial number of patients exhibit one or more persistent symptoms, either persisting from the acute phase of infection or re-emerging and lasting for an extended period [36]. In our study, most of the symptoms observed in the long-COVID-19 phase were already present during the acute phase, with only a very small percentage reporting new onset symptom.

The most frequently observed persistent symptom was fatigue, cognitive complaints, anxiety and depression, followed by dyspnea and headache. These findings are consistent with reports from the post-COVID-19 phase, where fatigue is the most frequently reported symptom [37,38,39,40,41,42,43,44], followed by dyspnea, headache, sleep disturbances, psychopathological and neurocognitive alterations such as memory, executive function and processing [10,11,12,37,45,46,47]. Additionally, anosmia and dysgeusia are primarily associated with the acute phase of the disease and tend to resolve in the post-COVID-19 phase [19].

When evaluating the evolution (pre-post) of patients with persistent symptoms at 6 months, we observed an overall cognitive improvement, with mild residual neuropsychological alterations regardless of the severity of illness, accompanied by the worsening of anxiety–depressive symptoms. These findings underscore a significant connection among psychopathological changes, the persistence of symptoms, and cognitive issues.

## 5. Limitations

These results must be considered within certain limitations. First, clinical information was collected through a retrospective review. The clinical symptoms observed at the onset of the disease and throughout the longitudinal follow-up, along with cognitive complaints, were gathered using open-ended questions that required yes or no responses. Similar studies in the future should use standardized questionnaires to document reported symptoms and should consider asking the participant if cognitive complaints are better, worse or had normalized. Additionally, a single self-report measure for anxiety and depression symptoms was also used. Future research should include more specific questionnaires to assess emotional functioning and sleep disturbances, as these have been widely shown to be related to poorer neurocognitive performance. Another limitation is the lack of parallel testing that would correct the learning effect.

A limitation that follows is the absence of other specific inflammatory biomarkers that may be linked to systemic inflammation such as IL-6, which has been widely implicated in these types of patients.

## 6. Conclusions

The results of this study allow us to describe the cognitive and clinical alterations observed at follow-up after COVID-19 infection. Persistent symptoms are frequently reported, including primarily fatigue, cognitive complaints, anxiety, and depression. Although cognition improves at follow-up, higher rates of anxiety and depression are associated with the presence of persistent symptoms. Patients with persistent symptoms 6 months post-infection should receive both psychopathological and cognitive evaluations.

## Figures and Tables

**Table 1:** Neuropsychological results: comparison between baseline and follow-up performance in the entire sample.

Neuropsychological Tests	Basal Mean (SD)	6 Months Mean (SD)	Sig. (2-Tailed)	d Cohen	Effect (r)
TAVEC-1	6.38 (1.76)	7.62 (1.88)	0.001 *	0.68	0.32
TAVECTotal	53.18 (8.75)	57.23 (9.54)	0.001 *	0.44	0.21
TAVEC-B	5.27 (1.62)	5.50 (1.64)	0.171	0.14	0.07
TAVEC-IMR	11.15 (2.60)	12.26 (2.78)	0.001 *	0.41	0.20
TAVEC-IMRSC	12.19 (2.46)	13.30 (2.52)	0.001 *	0.44	0.21
TAVEC-DFR	11.74 (2.85)	12.56 (2.94)	0.001 *	0.28	0.14
TAVEC-DFRSC	12.27 (2.65)	13.32 (2.53)	0.001 *	0.40	0.19
TAVEC-REC	14.99 (1.28)	15.23 (1.33)	0.126	0.18	0.09
WMS-IMR	34.74 (5.93)	36.32 (5.40)	0.001 *	0.27	0.13
WMS-DFR	28.08 (9.04)	31.21 (8.18)	0.001 *	0.36	0.17
Digits Forward	5.81 (1.15)	5.88 (1.21)	0.407	0.05	0.02
Digits Backward	4.31 (1.12)	4.81 (2.64)	0.055	0.24	0.12
Letter and Number	9.62 (2.43)	9.79 (2.33)	0.233	0.07	0.03
TMT-A	36.31 (15.21)	35.18 (17.13)	0.281	0.06	0.03
TMT-B	97.56 (60.48)	88.66 (49.81)	0.028 *	0.16	0.08
SDMT	42.63 (11.63)	44.63 (12.31)	0.001 *	0.16	0.08
Stroop Lecture	100.66 (18.71)	97.67 (20.75)	0.014 *	0.15	0.07
Stroop Color	65.86 (11.51)	64.83 (12.56)	0.168	0.08	0.04
Stroop Interference	38.86 (10.76)	38.77 (10.92)	0.882	0.00	0.00
Semantic Fluency	23.41 (6.14)	23.99 (6.49)	0.209	0.09	0.04
Phonetic Fluency	14.26 (4.69)	15.15 (4.82)	0.016 *	0.18	0.09
FCRO copy	33.04 (4.16)	33.09 (4.05)	0.907	0.01	0.00
Boston Naming Test	50.44 (6.59)	51.65 (6.37)	0.001 *	0.18	0.09
HAD Anxiety	7.43 (4.19)	7.43 (4.45)	1.000	0.00	0.00
HAD Depresion	5.08 (3.90)	5.05 (4.28)	0.909	0.00	0.00

Abbreviations: TAVEC-1, Test de Aprendizaje Verbal España Complutense Complutense learning 1; TAVECTotal, Test de Aprendizaje Verbal España Complutense Complutense sum of learning; TAVEC-B, Test de Aprendizaje Verbal España Complutense Complutense learning B; TAVEC-IMR, Test de Aprendizaje Verbal España Complutense Complutense Immediate Recall; TAVEC-IMRSC, Test de Aprendizaje Verbal España Complutense Complutense Immediate Recall Semantic Clue; TAVEC-DFR, Test de Aprendizaje Verbal España Complutense Deferred Free Recall; TAVEC-DFRSC, Test de Aprendizaje Verbal España Complutense Deferred Free Recall Semantic Clue; TAVEC-REC, Test de Aprendizaje Verbal España Complutense Recognition; WMS-IMR, Visual Reproduction of the Wechsler Memory Scale—IV Immediate Recall; WMS-DFR, Visual Reproduction of the Wechsler Memory Scale—IV Deferred Free Recall; TMT-A, Trail-Making Test A; TMT-B, Trail-Making Test B; SDMT, Symbol Digit Modalities Test; FCRO, Complex Figure of Rey-Osterrieth; HAD, Hospital Anxiety and Depression scale; SD, standard deviation; * Test with significant *p* value.

**Table 2:** Neuropsychological results: comparison between baseline and follow-up performance for patients without initial admission, NH (*n* = 10).

Neuropsychological Tests	Basal Mean (SD)	6 Months Mean (SD)	Sig. (2-Tailed)	d Cohen	Effect (r)
TAVEC-1	6.56 (1.59)	8.00 (1.58)	0.026 *	0.90	0.41
TAVECTotal	57.44 (6.63)	60.22 (7.19)	0.054	0.40	0.19
TAVEC-B	4.89 (1.05)	5.44 (1.66)	0.325	0.39	0.19
TAVEC-IMR	12.89 (2.14)	13.33 (1.93)	0.525	0.21	0.10
TAVEC-IMRSC	12.78 (2.27)	14.33 (1.41)	0.043 *	0.82	0.37
TAVEC-DFR	13.11 (2.14)	13.33 (1.58)	0.791	0.11	0.05
TAVEC-DFRSC	13.22 (1.92)	14.11 (1.16)	0.104	0.56	0.27
TAVEC-REC	14.89 (1.05)	15.56 (0.72)	0.022	0.74	0.34
WMS-IMR	35.78 (4.94)	37.11 (5.55)	0.207	0.25	0.12
WMS-DFR	30.44 (9.48)	31.56 (10.16)	0.531	0.11	0.05
Digits Forward	6.22 (0.83)	6.22 (1.20)	1.000	0.00	0.00
Digits Backward	4.78 (1.20)	5.11 (1.16)	0.195	0.27	0.13
Letter and Number	9.89 (1.83)	10.22 (1.71)	0.620	0.18	0.09
TMT-A	32.44 (10.16)	28.11 (5.46)	0.069	0.53	0.25
TMT-B	73.67 (21.98)	70.78 (15.97)	0.758	0.15	0.07
SDMT	47.67 (8.13)	49.33 (6.94)	0.233	0.21	0.10
Stroop Lecture	100.89 (12.59)	101.78 (11.30)	0.634	0.07	0.03
Stroop Color	65.44 (6.46)	65.56 (7.19)	0.958	0.01	0.00
Stroop Interference	39.33 (8.04)	38.33 (7.24)	0.629	0.13	0.06
Semantic Fluency	24.00 (3.20)	23.78 (4.08)	0.900	0.06	0.02
Phonetic Fluency	15.22 (4.29)	15.67 (2.55)	0.762	0.12	0.06
FCRO copy	34.38 (2.31)	34.77 (1.48)	0.301	0.20	0.10
Boston Naming Test	53.00 (5.85)	53.56 (5.00)	0.384	0.10	0.05
HAD Anxiety	10.33 (3.84)	7.67 (3.80)	0.007 *	0.69	0.32
HAD Depresion	7.33 (4.50)	5.22 (3.89)	0.106	0.50	0.24

Abbreviations:TAVEC-1, Test de Aprendizaje Verbal España Complutense learning 1; TAVECTotal, Test de Aprendizaje Verbal España Complutense sum of learning; TAVEC-B, Test de Aprendizaje Verbal España Complutense learning B; TAVEC-IMR, Test de Aprendizaje Verbal España Complutense Immediate Recall; TAVEC-IMRSC, Test de Aprendizaje Verbal España Complutense Immediate Recall Semantic Clue; TAVEC-DFR, Test de Aprendizaje Verbal España Complutense Deferred Free Recall; TAVEC-DFRSC, Test de Aprendizaje Verbal España Complutense Deferred Free Recall Semantic Clue; TAVEC-REC, Test de Aprendizaje Verbal España Complutense Recognition; WMS-IMR, Visual Reproduction of the Wechsler Memory Scale—IV Immediate Recall; WMS-DFR, Visual Reproduction of the Wechsler Memory Scale—IV Deferred Free Recall; TMT-A, Trail-Making Test A; TMT-B, Trail-Making Test B; SDMT, Symbol Digit Modalities Test; FCRO, Complex Figure of Rey-Osterrieth; HAD, Hospital Anxiety and Depression scale; SD, standard deviation; * Test with significant *p* value.

**Table 3:** Neuropsychological results: comparison between baseline and follow-up performance for patients admitted and without oxygen, HOSP (*n* = 21).

Neuropsychological Tests	Basal Mean (SD)	6 Months Mean (SD)	Sig. (2-Tailed)	d Cohen	Effect (r)
TAVEC-1	6.57 (1.43)	8.05 (1.71)	0.003 *	0.93	0.42
TAVECTotal	54.33 (6.60)	69.52 (9.25)	0.002 *	1.89	0.68
TAVEC-B	6.10 (1.48)	6.43 (1.53)	0.391	0.21	0.10
TAVEC-IMR	11.19 (2.48)	12.38 (2.67)	0.033 *	0.46	0.22
TAVEC-IMRSC	12.14 (2.08)	13.33 (2.03)	0.009 *	0.57	0.27
TAVEC-DFR	11.76 (2.56)	12.62 (3.07)	0.269	0.30	0.15
TAVEC-DFRSC	12.38 (2.03)	13.24 (2.44)	0.143	0.38	0.18
TAVEC-REC	15.14 (0.96)	14.62 (1.77)	0.248	0.36	0.17
WMS-IMR	35.76 (6.47)	36.67 (5.73)	0.247	0.14	0.07
WMS-DFR	29.29 (9.63)	32.71 (8.11)	0.071	0.39	0.18
Digits Forward	5.90 (1.09)	6.00 (1.14)	0.649	0.08	0.04
Digits Backward	4.43 (1.14)	4.71 (1.10)	0.208	0.24	0.12
Letter and Number	9.86 (2.22)	9.90 (2.14)	0.853	0.01	0.00
TMT-A	34.33 (11.82)	31.81 (11.78)	0.185	0.21	0.10
TMT-B	90.29 (67.31)	81.67 (55.71)	0.422	0.13	0.06
SDMT	46.05 (10.25)	46.05 (11.01)	1.000	0.00	0.00
Stroop Lecture	105.90 (15.81)	102.10 (21.76)	0.181	0.19	0.09
Stroop Color	68.95 (11.93)	68.29 (12.74)	0.689	0.05	0.02
Stroop Interference	43.00 (10.86)	43.00 (11.91)	1.000	0.00	0.00
Semantic Fluency	25.29 (5.33)	24.29 (5.78)	0.188	0.19	0.09
Phonetic Fluency	15.14 (4.99)	15.86 (5.03)	0.379	0.14	0.07
FCRO copy	32.69 (5.74)	32.92 (5.37)	0.763	0.04	0.02
Boston Naming Test	51.05 (6.31)	51.67 (6.12)	0.473	0.09	0.04
HAD Anxiety	7.48 (3.91)	6.90 (4.41)	0.463	0.13	0.06
HAD Depresion	5.19 (4.30)	4.24 (3.82)	0.212	0.23	0.11

Abbreviations: TAVEC-1, Test de Aprendizaje Verbal España Complutense learning 1; TAVECTotal, Test de Aprendizaje Verbal España Complutense sum of learning; TAVEC-B, Test de Aprendizaje Verbal España Complutense learning B; TAVEC-IMR, Test de Aprendizaje Verbal España Complutense Immediate Recall; TAVEC-IMRSC, Test de Aprendizaje Verbal España Complutense Immediate Recall Semantic Clue; TAVEC-DFR, Test de Aprendizaje Verbal España Complutense Deferred Free Recall; TAVEC-DFRSC, Test de Aprendizaje Verbal España Complutense Deferred Free Recall Semantic Clue; TAVEC-REC, Test de Aprendizaje Verbal España Complutense Recognition; WMS-IMR, Visual Reproduction of the Wechsler Memory Scale—IV Immediate Recall; WMS-DFR, Visual Reproduction of the Wechsler Memory Scale—IV Deferred Free Recall; TMT-A, Trail-Making Test A; TMT-B, Trail-Making Test B; SDMT, Symbol Digit Modalities Test; FCRO, Complex Figure of Rey-Osterrieth; HAD, Hospital Anxiety and Depression scale; SD, standard deviation; * Test with significant *p* value.

**Table 4:** Neuropsychological results: comparison between baseline and follow-up performance for patients with oxygen, OXY (*n* = 56).

Neuropsychological Tests	Basal Mean (SD)	6 Months Mean (SD)	Sig. (2-Tailed)	d Cohen	Effect (r)
TAVEC-1	6.34 (1.98)	7.61 (2.01)	0.001 *	0.63	0.30
TAVECTotal	52.77 (9.72)	56.39 (10.18)	0.002 *	0.36	0.17
TAVEC-B	5.20 (1.64)	5.20 (1.38)	1.000	0.00	0.00
TAVEC-IMR	10.88 (2.69)	11.96 (2.95)	0.001 *	0.38	0.18
TAVEC-IMRSC	12.04 (2.66)	12.98 (2.89)	0.003 *	0.33	0.16
TAVEC-DFR	11.52 (2.98)	12.41 (3.20)	0.008 *	0.28	0.14
TAVEC-DFRSC	11.96 (2.82)	13.14 (2.77)	0.001 *	0.42	0.20
TAVEC-REC	15.04 (1.25)	15.36 (1.18)	0.092	0.26	0.13
WMS-IMR	34.86 (5.39)	36.25 (5.23)	0.003 *	0.26	0.12
WMS-DFR	27.55 (9.27)	31.31 (8.29)	0.001 *	0.42	0.20
Digits Forward	5.82 (1.26)	5.80 (1.28)	0.878	0.01	0.00
Digits Backward	4.21 (1.09)	4.54 (0.97)	0.038 *	0.31	0.15
Letter and Number	9.86 (2.62)	9.84 (2.54)	0.932	0.00	0.00
TMT-A	36.20 (14.21)	36.20 (19.17)	1.000	0.00	0.00
TMT-B	98.64 (59.20)	93.64 (53.54)	0.353	0.08	0.04
SDMT	41.63 (12.21)	44.21 (12.56)	0.001 *	0.20	0.10
Stroop Lecture	100.89 (19.03)	97.69 (21.25)	0.084	0.15	0.07
Stroop Color	66.38 (10.40)	64.47 (13.25)	0.068	0.16	0.07
Stroop Interference	38.87 (10.71)	38.51 (11.10)	0.612	0.03	0.01
Semantic Fluency	23.16 (5.99)	24.61 (6.86)	0.035 *	0.22	0.11
Phonetic Fluency	13.86 (4.27)	15.07 (4.72)	0.022 *	0.26	0.13
FCRO copy	33.13 (3.24)	32.75 (3.79)	0.332	0.10	0.05
Boston Naming Test	50.38 (6.71)	51.61 (6.72)	0.001 *	0.18	0.09
HAD Anxiety	7.30 (4.43)	7.80 (4.79)	0.272	0.10	0.05
HAD Depresion	5.00 (3.81)	5.39 (4.69)	0.371	0.09	0.04

Abbreviations: TAVEC-1, Test de Aprendizaje Verbal España Complutense learning 1; TAVECTotal, Test de Aprendizaje Verbal España Complutense sum of learning; TAVEC-B, Test de Aprendizaje Verbal España Complutense learning B; TAVEC-IMR, Test de Aprendizaje Verbal España Complutense Immediate Recall; TAVEC-IMRSC, Test de Aprendizaje Verbal España Complutense Immediate Recall Semantic Clue; TAVEC-DFR, Test de Aprendizaje Verbal España Complutense Deferred Free Recall; TAVEC-DFRSC, Test de Aprendizaje Verbal España Complutense Deferred Free Recall Semantic Clue; TAVEC-REC, Test de Aprendizaje Verbal España Complutense Recognition; WMS-IMR, Visual Reproduction of the Wechsler Memory Scale—IV Immediate Recall; WMS-DFR, Visual Reproduction of the Wechsler Memory Scale—IV Deferred Free Recall; TMT-A, Trail-Making Test A; TMT-B, Trail-Making Test B; SDMT, Symbol Digit Modalities Test; FCRO, Complex Figure of Rey-Osterrieth; HAD, Hospital Anxiety and Depression scale; SD, standard deviation; * Test with significant *p* value.

**Table 5:** Neuropsychological results: comparison between baseline and follow-up performance for ICU patients (*n* = 21).

Neuropsychological Tests	Basal Mean (SD)	6 Months Mean (SD)	Sig. (2-Tailed)	d Cohen	Effect (r)
TAVEC-1	6.19 (1.63)	7.00 (1.76)	0.015 *	0.47	0.23
TAVECTotal	51.10 (8.55)	55.52 (8.84)	0.002 *	0.50	0.24
TAVEC-B	4.86 (1.71)	5.33 (2.10)	0.329	0.24	0.12
TAVEC-IMR	10.95 (2.53)	12.29 (2.70)	0.002 *	0.51	0.24
TAVEC-IMRSC	12.29 (2.47)	13.52 (2.25)	0.002 *	0.52	0.25
TAVEC-DFR	11.57 (3.02)	12.43 (2.61)	0.064	0.30	0.15
TAVEC-DFRSC	12.43 (2.97)	13.43 (2.39)	0.105	0.37	0.18
TAVEC-REC	14.71 (1.73)	15.33 (1.31)	0.148	0.40	0.19
WMS-IMR	32.86 (7.14)	35.86 (5.92)	0.032 *	0.45	0.22
WMS-DFR	26.95 (7.96)	29.10 (7.34)	0.202	0.28	0.13
Digits Forward	5.52 (1.03)	5.76 (1.17)	0.261	0.21	0.10
Digits Backward	4.24 (1.09)	5.52 (5.68)	0.316	0.31	0.15
Letter and Number	8.57 (2.22)	9.29 (2.61)	0.008 *	0.29	0.14
TMT-A	41.05 (21.30)	39.48 (18.50)	0.392	0.07	0.03
TMT-B	114.65 (67.63)	92.05 (43.75)	0.027 *	0.39	0.19
SDMT	39.43 (11.96)	42.24 (14.75)	0.052	0.20	0.10
Stroop Lecture	93.90 (21.75)	90.38 (20.74)	0.181	0.16	0.08
Stroop Color	61.30 (14.97)	61.55 (12.39)	0.899	0.01	0.00
Stroop Interference	34.25 (10.98)	34.90 (9.92)	0.670	0.06	0.03
Semantic Fluency	20.90 (6.06)	21.38 (5.90)	0.671	0.08	0.04
Phonetic Fluency	13.29 (4.49)	13.71 (4.60)	0.602	0.09	0.04
FCRO copy	32.42 (5.18)	32.92 (4.09)	0.368	0.10	0.05
Boston Naming Test	48.62 (6.80)	50.67 (6.46)	0.001 *	0.30	0.15
HAD Anxiety	6.67 (3.66)	7.05 (3.95)	0.662	0.09	0.04
HAD Depresion	4.43 (3.55)	5.10 (3.82)	0.379	0.18	0.09

Abbreviations: TAVEC-1, Test de Aprendizaje Verbal España Complutense learning 1; TAVECTotal, Test de Aprendizaje Verbal España Complutense sum of learning; TAVEC-B, Test de Aprendizaje Verbal España Complutense learning B; TAVEC-IMR, Test de Aprendizaje Verbal España Complutense Immediate Recall; TAVEC-IMRSC, Test de Aprendizaje Verbal España Complutense Immediate Recall Semantic Clue; TAVEC-DFR, Test de Aprendizaje Verbal España Complutense Deferred Free Recall; TAVEC-DFRSC, Test de Aprendizaje Verbal España Complutense Deferred Free Recall Semantic Clue; TAVEC-REC, Test de Aprendizaje Verbal España Complutense Recognition; WMS-IMR, Visual Reproduction of the Wechsler Memory Scale—IV Immediate Recall; WMS-DFR, Visual Reproduction of the Wechsler Memory Scale—IV Deferred Free Recall; TMT-A, Trail-Making Test A; TMT-B, Trail-Making Test B; SDMT, Symbol Digit Modalities Test; FCRO, Complex Figure of Rey-Osterrieth; HAD, Hospital Anxiety and Depression scale; SD, standard deviation; * Test with significant *p* value.

**Table 6:** Neuropsychological results: comparison between baseline and follow-up performance for the subgroup without SCC.

Neuropsychological Tests	Subjects without SCC (*n* = 70) Basal (SD)/6 Months (SD)		Sig. (2-Tailed)	d Cohen	Effect (r)
TAVEC-1	6.47 (1.85)	7.43 (1.85)	0.001 *	0.51	0.25
TAVECTotal	52.83 (9.37)	56.59 (10.12)	0.001 *	0.38	0.18
TAVEC-B	4.99 (1.48)	5.11 (1.69)	0.554	0.07	0.03
TAVEC-IMR	11.03 (2.66)	12.03 (2.89)	0.001 *	0.36	0.17
TAVEC-IMRSC	12.09 (2.64)	13.14 (2.69)	0.001 *	0.39	0.19
TAVEC-DFR	11.74 (3.03)	12.41 (3.19)	0.060	0.21	0.10
TAVEC-DFRSC	12.16 (2.83)	13.17 (2.74)	0.002 *	0.36	0.17
TAVEC-REC	14.97 (1.32)	15.26 (1.51)	0.204	0.20	0.10
WMS-IMR	35.33 (5.96)	37.11 (4.81)	0.001 *	0.32	0.16
WMS-DFR	29.38 (8.92)	31.59 (8.43)	0.006 *	0.25	0.12
Digits Forward	5.93 (1.22)	6.01 (1.25)	0.443	0.06	0.03
Digits Backward	4.40 (1.16)	4.60 (1.05)	0.118	0.18	0.09
Letter and Number	9.81 (2.51)	9.89 (2.36)	0.679	0.03	0.01
TMT-A	36.53 (16.92)	34.30 (18.23)	0.061	0.12	0.06
TMT-B	94.68 (54.06)	89.87 (53.84)	0.291	0.08	0.04
SDMT	43.31 (12.05)	44.54 (13.04)	0.076	0.09	0.04
Stroop Lecture	101.51 (18.98)	97.03 (21.43)	0.012 *	0.22	0.10
Stroop Color	66.28 (12.94)	64.50 (14.01)	0.090	0.13	0.06
Stroop Interference	39.54 (11.54)	38.53 (11.92)	0.155	0.08	0.04
Semantic Fluency	23.67 (5.88)	24.61 (6.48)	0.099	0.15	0.07
Phonetic Fluency	14.63 (4.65)	15.14 (4.98)	0.262	0.10	0.05
FCRO copy	33.13 (4.46)	33.35 (3.62)	0.537	0.05	0.02
Boston Naming Test	51.30 (6.51)	52.21 (6.21)	0.005 *	0.14	0.07
HAD Anxiety	5.89 (3.54)	6.26 (4.32)	0.307	0.09	0.04
HAD Depresion	3.59 (3.08)	4.10 (3.97)	0.178	0.14	0.07

Abbreviations: TAVEC-1, Test de Aprendizaje Verbal España Complutense learning 1; TAVECTotal, Test de Aprendizaje Verbal España Complutense sum of learning; TAVEC-B, Test de Aprendizaje Verbal España Complutense learning B; TAVEC-IMR, Test de Aprendizaje Verbal España Complutense Immediate Recall; TAVEC-IMRSC, Test de Aprendizaje Verbal España Complutense Immediate Recall Semantic Clue; TAVEC-DFR, Test de Aprendizaje Verbal España Complutense Deferred Free Recall; TAVEC-DFRSC, Test de Aprendizaje Verbal España Complutense Deferred Free Recall Semantic Clue; TAVEC-REC, Test de Aprendizaje Verbal España Complutense Recognition; WMS-IMR, Visual Reproduction of the Wechsler Memory Scale—IV Immediate Recall; WMS-DFR, Visual Reproduction of the Wechsler Memory Scale—IV Deferred Free Recall; TMT-A, Trail-Making Test A; TMT-B, Trail-Making Test B; SDMT, Symbol Digit Modalities Test; FCRO, Complex Figure of Rey-Osterrieth; HAD, Hospital Anxiety and Depression scale; SD, standard deviation; * Test with significant *p* value.

**Table 7:** Neuropsychological results: comparison between baseline and follow-up performance for the subgroup with SCC.

Neuropsychological Tests	Subjects with SCC (*n* = 38) Basal (SD)/6 Mesos (SD)		Sig. (2-Tailed)	d Cohen	Effect (r)
TAVEC-1	6.27 (1.62)	7.95 (1.92)	0.001 *	0.94	0.42
TAVECTotal	53.73 (7.64)	58.24 (8.41)	0.001 *	0.56	0.27
TAVEC-B	5.84 (1.74)	6.19 (1.30)	0.186	0.22	0.11
TAVEC-IMR	11.30 (2.51)	12.59 (2.51)	0.001 *	0.51	0.24
TAVEC-IMRSC	12.32 (2.13)	13.51 (2.19)	0.001 *	0.55	0.26
TAVEC-DFR	11.65 (2.50)	12.76 (2.42)	0.001 *	0.45	0.22
TAVEC-DFRSC	12.41 (2.29)	13.54 (2.07)	0.001 *	0.51	0.25
TAVEC-REC	15.00 (1.22)	15.16 (0.92)	0.362	0.14	0.07
WMS-IMR	33.57 (5.85)	34.84 (6.23)	0.054	0.21	0.10
WMS-DFR	25.49 (8.87)	30.38 (7.82)	0.001 *	0.58	0.28
Digits Forward	5.59 (1.01)	5.59 (1.11)	1.000	0.00	0.00
Digits Backward	4.14 (1.05)	5.22 (4.29)	0.136	0.34	0.17
Letter and Number	9.22 (2.28)	9.57 (2.31)	0.156	0.15	0.07
TMT-A	36.35 (11.45)	37.19 (14.97)	0.693	0.06	0.03
TMT-B	104.06 (72.35)	87.00 (42.22)	0.036 *	0.28	0.14
SDMT	41.16 (10.94)	44.78 (11.14)	0.001 *	0.32	0.16
Stroop Lecture	98.62 (18.34)	98.27 (19.64)	0.743	0.01	0.00
Stroop Color	65.05 (8.60)	65.27 (9.66)	0.814	0.02	0.01
Stroop Interference	37.59 (9.34)	39.03 (9.02)	0.137	0.15	0.07
Semantic Fluency	22.32 (5.62)	22.38 (5.85)	0.947	0.01	0.00
Phonetic Fluency	13.14 (3.93)	14.76 (3.88)	0.011 *	0.41	0.20
FCRO copy	32.78 (3.60)	32.31 (4.78)	0.318	0.11	0.05
Boston Naming Test	48.65 (6.48)	50.43 (6.64)	0.001 *	0.27	0.13
HAD Anxiety	10.46 (3.69)	9.76 (3.78)	0.322	0.18	0.09
HAD Depresion	8.03 (3.65)	6.97 (4.25)	0.084	0.26	0.13

Abbreviations: TAVEC-1, Test de Aprendizaje Verbal España Complutense learning 1; TAVECTotal, Test de Aprendizaje Verbal España Complutense sum of learning; TAVEC-B, Test de Aprendizaje Verbal España Complutense learning B; TAVEC-IMR, Test de Aprendizaje Verbal España Complutense Immediate Recall; TAVEC-IMRSC, Test de Aprendizaje Verbal España Complutense Immediate Recall Semantic Clue; TAVEC-DFR, Test de Aprendizaje Verbal España Complutense Deferred Free Recall; TAVEC-DFRSC, Test de Aprendizaje Verbal España Complutense Deferred Free Recall Semantic Clue; TAVEC-REC, Test de Aprendizaje Verbal España Complutense Recognition; WMS-IMR, Visual Reproduction of the Wechsler Memory Scale—IV Immediate Recall; WMS-DFR, Visual Reproduction of the Wechsler Memory Scale—IV Deferred Free Recall; TMT-A, Trail-Making Test A; TMT-B, Trail-Making Test B; SDMT, Symbol Digit Modalities Test; FCRO, Complex Figure of Rey-Osterrieth; HAD, Hospital Anxiety and Depression scale; SD, standard deviation; * Test with significant *p* value.

## Data Availability

The data presented in this study are available on request from the corresponding author due to ethical reasons.

## Supplementary Materials

The following supporting information can be downloaded at: https://www.mdpi.com/article/10.3390/neurolint16040064/s1, Table S1: Neuropsychological results for Group 0 (No-No patients; *n* = 54). Table S2: Neuropsychological results for Group 1 (Yes-No patients; *n* = 16). Table S3: Neuropsychological results for Group 2 (No-Yes patients; *n* = 16). Table S4: Neuropsychological results for Group 3 (Yes-Yes patients; *n* = 22). Table S5: Neurocognitive subtests with differences in neuropsychological performance by four subgroups were defined based on the transition between baseline assessment and the 6-month follow-up. Table S6: Association between persistent symptoms and cognitive complaints.
