# Supplementary material for: Long Neurocognitive and Neuropsychiatric Sequelae in Participants with Post-COVID-19 Infection: A Longitudinal Study
Source: Neurol Int. 2024 Aug 16;16(4):853–68. doi: 10.3390/neurolint16040064 (PMC11357167; doi:10.3390/neurolint16040064)
Supplement: Supplementary file 1 [file neurolint-16-00064-s001.zip › neurolint-3137657-supplementary.pdf]

# Long Neurocognitive and Neuropsychiatric Sequelae in participants with post-COVID-19 infection: A longitudinal study

### Supplementary Materials

### Supplementary Tables

**Table S1.** Neuropsychological results for Group 0 (No-No patients; n=54)

| Neuropsychological Tests | Basal Mean (SD) | 6 months Mean (SD) | Sig. (2-tailed) | d Cohen | Effect (r) | Mean T Note 6 months (SD) |
|---|---|---|---|---|---|---|
| TAVEC-1 | 6.35 (1.58) | 7.67 (1.89) | 0.001* | 0.75 | 0.35 | 51.50 (8.85) |
| TAVECTotal | 54.09 (8.12) | 58.80 (8.32) | 0.001* | 0.57 | 0.27 | 58.80 (8.32) |
| TAVEC-B | 5.02 (1.42) | 5.26 (1.76) | 0.336 | 0.15 | 0.07 | 45.00 (8.41) |
| TAVEC-IMR | 11.39 (2.43) | 12.70 (2.56) | 0.001* | 0.52 | 0.25 | 56.66 (9.31) |
| TAVEC-IMRSC | 12.56 (2.43) | 13.63 (2.27) | 0.001* | 0.43 | 0.21 | 56.48 (9.93) |
| TAVEC-DFR | 12.15 (2.69) | 13.15 (2.49) | 0.002* | 0.38 | 0.18 | 56.29 (9.57) |
| TAVEC-DFRSC | 12.69 (2.57) | 13.62 (2.24) | 0.001* | 0.38 | 0.18 | 56.11 (9.79) |
| TAVEC-REC | 15.07 (1.33) | 15.46 (1.04) | 0.039* | 0.32 | 0.16 | 56.22 (5.62) |
| WMS-IMR | 35.78 (5.77) | 37.78 (4.55) | 0.001* | 0.38 | 0.18 | 50.84 (5.71) |
| WMS-DFR | 29.94 (9.10) | 32.87 (8.01) | 0.001* | 0.34 | 0.16 | 53.10 (6.15) |
| Digits Forward | 6.04 (1.19) | 6.22 (1.14) | 0.142 | 0.15 | 0.07 | 50.02 (6.58) |
| Digits Backward | 4.48 (1.12) | 4.63 (0.95) | 0.289 | 0.14 | 0.07 | 49.35 (5.43) |
| Letter&Number | 10.22 (2.40) | 10.33 (2.23) | 0.585 | 0.04 | 0.02 | 47.63 (6.64) |
| TMT-A | 34.09 (11.99) | 31.28 (12.93) | 0.004* | 0.22 | 0.11 | 49.58 (9.00) |
| TMT-B | 90.58 (51.14) | 80.02 (41.02) | 0.002* | 1.00 | 0.66 | 46.32 (7.57) |
| SDMT | 45.07 (11.55) | 46.65 (12.37) | 0.029* | 0.13 | 0.06 | 45.41 (7.10) |
| Stroop Lecture | 103.49 (16.79) | 101.06 (19.12) | 0.054 | 0.13 | 0.06 | 45.42 (7.35) |
| Stroop Color | 67.79 (12.80) | 67.15 (12.98) | 0.462 | 0.04 | 0.02 | 45.09 (7.03) |
| Stroop Int. | 40.63 (11.99) | 40.44 (11.62) | 0.754 | 0.01 | 0.00 | 44.51 (9.41) |
| Semantic Fluency | 24.44 (5.59) | 25.81 (6.21) | 0.026* | 0.02 | 0.11 | 51.01 (7.65) |
| Phonetic Fluency | 14.93 (4.55) | 15.80 (4.89) | 0.082 | 0.18 | 0.09 | 45.60 (6.60) |
| FCRO copy | 33.25 (4.66) | 33.45 (3.63) | 0.063 | 0.04 | 0.02 | 53.47 (9.42) |
| BNT | 51.46 (6.59) | 52.43 (6.54) | 0.002* | 0.14 | 0.07 | 49.75 (9.08) |
| HAD Anxiety | 5.37 (3.42) | 5.24 (3.67) | 0.404 | 0.03 | 0.01 | |
| HAD Depresion | 3.11 (2.95) | 3.31 (3.24) | 0.347 | 0.06 | 0.03 | |

Abbreviations: TAVEC-1, Test de Aprendizaje Verbal España Complutense learning 1; TAVECTotal, Test de Aprendizaje Verbal España Complutense sum of learning; TAVEC-B, Test de Aprendizaje Verbal España Complutense learning B; TAVEC-IMR, Test de Aprendizaje Verbal España Complutense Immediate Recall; TAVEC-IMRSC, Test de Aprendizaje Verbal España Complutense Immediate Recall Semantic Clue; TAVEC-DFR, Test de Aprendizaje Verbal España Complutense Deferred Free Recall; TAVEC-DFRSC, Test de Aprendizaje Verbal España Complutense Deferred Free Recall Semantic Clue; TAVEC-REC, Test de Aprendizaje Verbal España Complutense Recognition; WMS-IMR, Visual Reproduction of the Wechsler Memory Scale—IV Immediate Recall; WMS-DFR, Visual Reproduction of the Wechsler Memory Scale—IV Deferred Free Recall; TMT-A, Trail-Making Test A; TMT-B, Trail-Making Test B; SDMT, Symbol Digit Modalities Test; FCRO, Complex Figure of Rey-Osterrieth; HAD, Hospital Anxiety and Depression scale; SD, standard deviation; * Test with significant *p* value.

**Table S2.** Neuropsychological results for Group 1 (Yes-No patients; $n$ = 16)

| Neuropsychological Tests | Basal Mean (SD) | 6 months Mean (SD) | Sig. (2-tailed) | d Cohen | Effect (r) | Mean T Note 6 months (SD) |
|---|---|---|---|---|---|---|
| TAVEC-1 | 6.20 (1.69) | 8.07 (1.79) | 0.006* | 1.00 | 0.47 | 54.00 (9.85) |
| TAVECTotal | 53.53 (8.15) | 59.20 (7.79) | 0.010* | 0.71 | 0.33 | 59.20 (7.79) |
| TAVEC-B | 5.80 (2.07) | 6.20 (1.37 ) | 0.373 | 0.22 | 0.11 | 48.00 (8.61) |
| TAVEC-IMR | 11.40 (2.32) | 12.67 (2.25) | 0.022* | 0.55 | 0.26' | 56.00 (9.10) |
| TAVEC-IMRSC | 12.40 (1.88) | 13.80 (2.39) | 0.018* | 0.65 | 0.30 | 58.00 (8.61) |
| TAVEC-DFR | 11.27 (2.31) | 13.20 (2.04) | 0.001* | 0.88 | 0.40 | 55.33 (8.33) |
| TAVEC-DFRSC | 12.40 (1.92) | 13.87 (2.03) | 0.008* | 0.74 | 0.34 | 56.00 (9.10) |
| TAVEC-REC | 15.00 (1.36) | 15.47 (0.74) | 0.187 | 0.42 | 0.20 | 56.00 (5.07) |
| WMS-IMR | 34.60 (6.41) | 35.47 (6.15) | 0.474 | 0.13 | 0.06 | 46.66 (8.94) |
| WMS-DFR | 27.33 (10.08) | 30.67 (7.51) | 0.183 | 0.37 | 0.18 | 49.83 (8.31) |
| Digits Forward | 5.73 (1.28) | 5.87 (1.18) | 0.499 | 0.11 | 0.05 | 46.83 (7.22) |
| Digits Backward | 4.20 (1.20) | 6.33 (6.64) | 0.235 | 0.44 | 0.21 | 47.50 (7.07) |
| Letter&Number | 9.73 (2.46) | 9.60 (2.32) | 0.685 | 0.05 | 0.02 | 44.16 (6.52) |
| TMT-A | 37.27 (12.07) | 33.20 (8.57) | 0.133 | 0.38 | 0.19 | 44.33 (6.01) |
| TMT-B | 99.00 (79.48) | 86.20 (49.03) | 0.248 | 0.19 | 0.09 | 39.26 (11.65) |
| SDMT | 44.20 (10.33) | 47.47 (9.73) | 0.015* | 0.32 | 0.16 | 43.33 (7.17) |
| Stroop Lecture | 109.33 (18.42) | 110.80 (17.03) | 0.254 | 0.08 | 0.04 | 47.83 (8.49) |
| Stroop Color | 65.93 (9.53) | 67.93 (9.32) | 0.192 | 0.21 | 0.10 | 43.66 (5.96) |
| Stroop Int. | 38.00 (12.03) | 40.39 (10.30) | 0.101 | 0.21 | 0.10 | 43.83 (7.37) |
| Semantic Fluency | 22.53 (6.97) | 22.60 (6.03) | 0.964 | 0.01 | 0.00 | 45.20 (8.64) |
| Phonetic Fluency | 13.27 (4.84) | 14.60 (3.94) | 0.126 | 0.30 | 0.14 | 42.33 (6.71) |
| FCRO copy | 33.23 (4.27) | 32.50 (5.25) | 0.380 | 0.15 | 0.07 | 51.50 (9.76) |
| BNT | 48.07 (7.32) | 50.13 (7.41) | 0.003* | 0.27 | 0.13 | 44.33 (8.78) |
| HAD Anxiety | 10.27 (3.28) | 7.33 (2.12) | 0.004* | 1.00 | 0.46 | |
| HAD Depresion | 7.07 (3.41) | 4.20 (2.93) | 0.016* | 0.90 | 0.41 | |

Abbreviations: TAVEC-1, Test de Aprendizaje Verbal España-Complutense learning 1; TAVECTotal, Test de Aprendizaje Verbal España-Complutense sum of learning; TAVEC-B, Test de Aprendizaje Verbal España-Complutense learning B; TAVEC-IMR, Test de Aprendizaje Verbal España-Complutense Immediate Recall; TAVEC-IMRSC, Test de Aprendizaje Verbal España-Complutense Immediate Recall Semantic Clue; TAVEC-DFR, Test de Aprendizaje Verbal España-Complutense Deferred Free Recall; TAVEC-DFRSC, Test de Aprendizaje Verbal España-Complutense Deferred Free Recall Semantic Clue; TAVEC-REC, Test de Aprendizaje Verbal España-Complutense Recognition; WMS-IMR, Visual Reproduction of the Wechsler Memory Scale –IV Immediate Recall; WMS-DFR, Visual Reproduction of the Wechsler Memory Scale –IV Deferred Free Recall; Int, Intereference; TMT-A, Trail Making Test A; TMT-B, Trail Making Test B; SDMT, Symbol Digit Modalities Test; FCRO, Complex Figure of Rey-Osterrieth; BNT, Boston Naming Test; HAD, Hospital Anxiety and Depression scale; SD, standard deviation. * Test with significant $p$ value.

**Table S3.** Neuropsychological results for Group 2 (No-Yes patients; $n$ = 16)

| Neuropsychological Tests | Basal Mean (SD) | 6 months Mean (SD) | Sig. (2-tailed) | d Cohen | Effect (r) | Mean T Note 6 months (SD) |
|---|---|---|---|---|---|---|
| TAVEC-1 | 6.69 (2.62) | 6.63 (1.50) | 0.916 | 0.02 | 0.01 | 47.50 (7.74) |
| TAVECTotal | 48.56 (12.07) | 49.13 (12.25) | 0.841 | 0.04 | 0.02 | 49.13 (12.25) |
| TAVEC-B | 4.88 (1.74) | 4.63 (1.36) | 0.580 | 0.16 | 0.07 | 43.12 (7.04) |
| TAVEC-IMR | 9.81 (3.10) | 9.75 (2.88) | 0.931 | 0.02 | 0.01 | 45.00 (9.66) |
| TAVEC-IMRSC | 10.50 (2.75) | 11.50 (3.36) | 0.269 | 0.32 | 0.16 | 49.37 (12.36) |
| TAVEC-DFR | 10.38 (3.75) | 9.94 (4.05) | 0.704 | 0.11 | 0.05 | 46.00 (14.04) |
| TAVEC-DFRSC | 10.38 (3.00) | 11.31 (3.47) | 0.398 | 0.28 | 0.14 | 48.66 (12.45) |
| TAVEC-REC | 14.63 (1.31) | 14.56 (2.44) | 0.936 | 0.03 | 0.01 | 55.00 (5.18) |
| WMS-IMR | 33.81 (6.51) | 34.88 (5.16) | 0.435 | 0.18 | 0.09 | 47.50 (5.08) |
| WMS-DFR | 27.50 (8.30) | 27.38 (8.67) | 0.956 | 0.01 | 0.00 | 49.06 (5.97) |
| Digits Forward | 5.56 (1.26) | 5.31 (1.40) | 0.232 | 0.18 | 0.09 | 50.62 (6.55) |
| Digits Backward | 4.13 (1.25) | 4.50 (1.36) | 0.849 | 0.28 | 0.14 | 44.53 (4.84) |
| Letter&Number | 8.44 (2.42) | 8.38 (2.21) | 0.952 | 0.02 | 0.01 | 45.93 (6.44) |
| TMT-A | 44.75 (26.76) | 44.50 (28.19) | 0.358 | 0.00 | 0.00 | 41.56 (5.76) |
| TMT-B | 109.13 (63.11) | 124.67 (77.17) | 1.000 | 0.22 | 0.10 | 42.81 (4.81) |
| SDMT | 37.38 (12.15) | 37.38 (13.09) | 0.086 | 0.00 | 0.00 | 41.25 (5.91) |
| Stroop Lecture | 94.94 (24.42) | 83.69 (23.80) | 0.118 | 0.46 | 0.22 | 40.96 (6.01) |
| Stroop Color | 61.38 (12.53) | 55.88 (14.15) | 0.111 | 0.41 | 0.20 | 42.65 (5.80) |
| Stroop Int. | 36.00 (9.38) | 32.31 (11.03) | 0.727 | 0.36 | 0.17 | 45.62 (7.98) |
| Semantic Fluency | 21.06 (6.28) | 20.56 (5.86) | 0.531 | 0.08 | 0.04 | 43.43 (7.23) |
| Phonetic Fluency | 13.63 (4.99) | 12.94 (4.80) | 0.531 | 0.14 | 0.07 | 43.43 (7.23) |
| FCRO copy | 32.71 (3.82) | 33.00 (3.69) | 0.669 | 0.07 | 0.03 | 53.28 (9.2) |
| BNT | 50.75 (6.44) | 51.50 (5.06) | 0.449 | 0.12 | 0.06 | 49.21 (6.75) |
| HAD Anxiety | 7.63 (3.48) | 9.69 (4.71) | 0.007* | 0.49 | 0.24 | |
| HAD Depresion | 5.19 (3.06) | 6.75 (5.06) | 0.197 | 0.37 | 0.18 | |

Abbreviations: TAVEC-1, Test de Aprendizaje Verbal España-Complutense learning 1; TAVECTotal, Test de Aprendizaje Verbal España-Complutense sum of learning; TAVEC-B, Test de Aprendizaje Verbal España-Complutense learning B; TAVEC-IMR, Test de Aprendizaje Verbal España-Complutense Immediate Recall; TAVEC-IMRSC, Test de Aprendizaje Verbal España-Complutense Immediate Recall Semantic Clue; TAVEC-DFR, Test de Aprendizaje Verbal España-Complutense Deferred Free Recall; TAVEC-DFRSC, Test de Aprendizaje Verbal España-Complutense Deferred Free Recall Semantic Clue; TAVEC-REC, Test de Aprendizaje Verbal España-Complutense Recognition; WMS-IMR, Visual Reproduction of the Wechsler Memory Scale –IV Immediate Recall; WMS-DFR, Visual Reproduction of the Wechsler Memory Scale –IV Deferred Free Recall; Int, Intereference; TMT-A, Trail Making Test A; TMT-B, Trail Making Test B; SDMT, Symbol Digit Modalities Test; FCRO, Complex Figure of Rey-Osterrieth; BNT, Boston Naming Test; HAD, Hospital Anxiety and Depression scale; SD, standard deviation. * Test with significant $p$ value.

**Table S4.** Neuropsychological results for Group 3 (Yes-Yes patients; *n*=22).

| **Neuropshycological Tests** | **Basal Mean (SD)** | **6 months Mean (SD)** | **Sig. (2-tailed)** | **d Cohen** | **Effect (r)** | **Mean T note 6 months (SD)** |
|---|---|---|---|---|---|---|
| TAVEC-1 | 6.32 (1.61) | 7.83 (2.05) | 0.001* | 0.81 | 0.37 | 52.27 (10.66) |
| TAVECTotal | 53.86 (7.46) | 57.59 (8.93) | 0.007* | 0.45 | 0.22 | 57.59 (8.93) |
| TAVEC-B | 5.86 (1.58) | 6.18 (1.29) | 0.348 | 0.22 | 0.11 | 48.63 (6.39) |
| TAVEC-IMR | 11.23 (2.68) | 12.55 (2.72) | 0.001* | 0.48 | 0.23 | 53.63 (10.02) |
| TAVEC-IMRSC | 12.27 (2.33) | 13.32 (2.07) | 0.006* | 0.47 | 0.23 | 54.54 (8.57) |
| TAVEC-DFR | 11.91 (2.65) | 12.45 (2.65) | 0.049* | 0.20 | 0.10 | 52.27 (10.66) |
| TAVEC-DFRSC | 12.41 (2.55) | 13.32 (2.12) | 0.008* | 0.38 | 0.19 | 54.09 (9.08) |
| TAVEC-REC | 15.00 (1.15) | 14.95 (0.99) | 0.803 | 0.04 | 0.02 | 50.90 (9.21) |
| WMS-IMR | 32.86 (5.48) | 34.41 (6.40) | 0.047* | 0.26 | 0.12 | 46.13 (8.88) |
| WMS-DFR | 24.23 (7.94) | 30.18 (8.19) | 0.001* | 0.73 | 0.34 | 50.45 (6.97) |
| Digits Forward | 5.50 (0.80) | 5.41 (1.05) | 0.665 | 0.09 | 0.04 | 44.77 (6.72) |
| Digits Backward | 4.09 (0.97) | 4.45 (0.85) | 0.104 | 0.39 | 0.19 | 48.29 (5.84) |
| Letter&Number | 8.86 (2.14) | 9.55 (2.36) | 0.052 | 0.30 | 0.15 | 46.02 (6.10) |
| TMT-A | 35.73 (11.26) | 39.91 (17.80) | 0.167 | 0.28 | 0.13 | 44.88 (9.92) |
| TMT-B | 107.85 (69.38) | 87.60 (37.66) | 0.090 | 0.36 | 0.17 | 43.37 (8.24) |
| SDMT | 39.09 (11.09) | 42.95 (11.87) | 0.008* | 0.33 | 0.16 | 43.29 (6.51) |
| Stroop Lecture | 91.32 (14.58) | 89.73 (16.71) | 0.315 | 0.10 | 0.05 | 40.11 (6.96) |
| Stroop Color | 64.45 (8.08) | 63.45 (9.68) | 0.385 | 0.11 | 0.05 | 42.61 (6.29) |
| Stroop Int. | 37.32 (7.27) | 37.73 (8.03) | 0.709 | 0.05 | 0.02 | 43.29 (7.29) |
| Semantic Fluency | 22.18 (4.65) | 22.23 (5.87) | 0.963 | 0.00 | 0.00 | 46.81 (7.56) |
| Phonetic Fluency | 13.05 (3.30) | 14.86 (3.93) | 0.049* | 0.49 | 0.24 | 44.43 (6.12) |
| FCRO copy | 32.47 (3.13) | 32.18 (4.55) | 0.611 | 0.07 | 0.03 | 51.00 (10.52) |
| BNT | 49.05 (5.99) | 50.64 (6.23) | 0.009* | 0.26 | 0.12 | 46.68 (9.04) |
| HAD Anxiety | 10.59 (4.02) | 11.41 (3.80) | 0.373 | 0.20 | 0.10 | |
| HAD Depresion | 8.68 (3.74) | 8.86 (4.00) | 0.757 | 0.04 | 0.02 | |

Abbreviations: TAVEC-1, Test de Aprendizaje Verbal España-Complutense learning 1; TAVECTotal, Test de Aprendizaje Verbal España-Complutense sum of learning; TAVEC-B, Test de Aprendizaje Verbal España-Complutense learning B; TAVEC-IMR, Test de Aprendizaje Verbal España-Complutense Immediate Recall; TAVEC-IMRSC, Test de Aprendizaje Verbal España-Complutense Immediate Recall Semantic Clue; TAVEC-DFR, Test de Aprendizaje Verbal España-Complutense Deferred Free Recall; TAVEC-DFRSC, Test de Aprendizaje Verbal España-Complutense Deferred Free Recall Semantic Clue; TAVEC-REC, Test de Aprendizaje Verbal España-Complutense Recognition; WMS-IMR, Visual Reproduction of the Wechsler Memory Scale –IV Immediate Recall; WMS-DFR, Visual Reproduction of the Wechsler Memory Scale –IV Deferred Free Recall; Int, Intereference; TMT-A, Trail Making Test A; TMT-B, Trail Making Test B; SDMT, Symbol Digit Modalities Test; FCRO, Complex Figure of Rey-Osterrieth; BNT, Boston Naming Test; HAD, Hospital Anxiety and Depression scale; SD, standard deviation. * Test with significant *p* value.

**Table S5.** Neurocognitive subtests with differences in neuropsychological performance by four subgroups were defined based on the transition between baseline assessment and the 6-month follow-up.

| NPS Tests | ANOVA F | Group 0-1 (sig). | Group 0-2 (sig.) | Group 0-3 (sig.) | Group 1-2 (sig.) | Group 1-3 (sig.) | Group 2-3 (sig.) |
|---|---|---|---|---|---|---|---|
| TAVEC Total | 5.05 | - | 0.004 | - | 0.027 | - | - |
| TAVEC-IMR | 5.67 | - | 0.002 | - | 0.025 | - | 0.017 |
| TAVEC-IMRSC | 3.42 | - | 0.002 | - | - | - | - |
| TAVEC-DFR | 5.93 | - | 0.001 | - | - | - | - |
| TAVEC-DFRSC | 4.43 | - | 0.009 | - | 0.039 | - | - |
| Letter&Numbers | 3.22 | - | 0.003 | - | - | - | - |
| TMT-B | 5.11 | - | 0.003 | - | - | - | 0.048 |
| Stroop Lecture | 6.99 | - | 0.002 | - | 0.002 | 0.016 | - |
| Stroop Color | 3.98 | - | 0.001 | - | - | - | - |
| Semantic F. | 4.18 | - | 0.003 | - | - | - | - |
| HAD Anxiety | 16.72 | - | 0.001 | 0.001 | - | 0.016 | - |
| HAD Depresion | 13.23 | - | 0.017 | 0.001 | - | 0.004 | - |

Abbreviations: TavecTotal, Test de Aprendizaje Verbal España-Complutense sum of learning; TAVEC-IMR, Test de Aprendizaje Verbal España-Complutense Immediate Recall; TAVEC-IMRSC, Test de Aprendizaje Verbal España-Complutense Immediate Recall Semantic Clue; TAVEC-DFR, Test de Aprendizaje Verbal España-Complutense Deferred Free Recall; TAVEC-DFRSC, Test de Aprendizaje Verbal España-Complutense Deferred Free Recall Semantic Clue; TMT-B, Trail Making Test B; F, fluency; HAD, Hospital Anxiety and Depression scale; NPS, Neuropsychological.

**Table S6.** Association between persistent symptoms and cognitive complaints

| | Without Persistent Symptoms | With Persistent Symptoms |
|---|---|---|
| **Group 0 (No-No)** | *n* = 39 (72.2%) | *n* = 15 (27.8%) |
| **Group 1 (Yes-No)** | *n* = 12 (75%) | *n* = 4 (25%) |
| **Group 2 (No-Yes)** | *n* = 4 (25%) | *n* = 12 (75%) |
| **Group 3 (Yes-Yes)** | *n* = 5 (22.7%) | *n* = 17 (77.3%) |
